# Comparative efficacy of Jaungo, a traditional herbal ointment, and a water-in-oil type non-steroidal moisturizer for radiation-induced dermatitis in patients with breast cancer: a prospective, randomized, single-blind, pilot study

**DOI:** 10.3389/fphar.2023.1216668

**Published:** 2023-07-03

**Authors:** Eun Hye Kim, Su Bin Park, Hayun Jin, Weon Kuu Chung, Seong Woo Yoon

**Affiliations:** ^1^ Department of Clinical Korean Medicine, College of Korean Medicine, Graduate School, Kyung Hee University, Seoul, Republic of Korea; ^2^ Department of Korean Internal Medicine, Kyung Hee University Hospital at Gangdong, Seoul, Republic of Korea; ^3^ Department of Radiation Oncology, Kyung Hee University of Gangdong, Seoul, Republic of Korea

**Keywords:** Jaungo, Shiunko, radiation-induced dermatitis, traditional medicine, breast cancer

## Abstract

**Background:** Radiation-induced dermatitis (RID) is one of the most prevalent side-effects of conventional cancer therapies; however, there is no standard treatment for its prevention. Therefore, this study aimed to evaluate the comparative efficacy and safety of Jaungo (mainly composed of *Lithospermum erythrorhizon* Siebold & Zucc. and *Angelica sinensis* (Oliv.) Diel) and the water-in-oil-type non-steroidal moisturizer for the prevention of RID in patients with breast cancer (BC).

**Methods:** This is a prospective, single-blind, pilot randomized controlled trial. Between March 2021 and July 2022, 50 patients were randomly selected to use Jaungo or the moisturizer while undergoing postoperative radiation therapy (RT). Assessments were conducted nine times—every week while the patients underwent RT and 2 weeks after the end of therapy. The primary outcome was the incidence rate of RID grade ≥2. The secondary outcomes were the incidence rate of maximum grade RID, time to RID onset, RID-related quality of life (QOL) score, pain intensity, and adverse events.

**Results:** The incidence rate of RID grade ≥2 was 24.0% and 20.0% in the Jaungo and the moisturizer groups, respectively, with no significant difference between the groups (*p* = 0.733). Regarding the secondary outcomes, the incidence rate of maximum grade RID (*p* = 0.890), mean time to RID onset (*p* = 0.092), cumulative incidence rate of RID (*p* = 0.280), RID-related QOL score, and maximum pain intensity (*p* = 0.844) also did not differ significantly between the groups. None of the subjects in either group experienced severe adverse effects, although one participant in the moisturizer group had mild fever and insomnia.

**Conclusion:** These findings suggest that Jaungo has preventive efficacy without severe side-effects against RID in patients with BC that is comparable to that of the water-in-oil type non-steroidal moisturizer. Further extensive randomized controlled trials with larger sample sizes are warranted to validate our findings.

**Clinical Trial Registration:** Clinical Research Information Service (CRIS), https://cris.nih.go.kr, identifier KCT0005971.

## 1 Introduction

Radiation therapy (RT) is one of the most common conventional therapies for patients with cancer (Bolderston et al., 2006). More than 70% of patients with breast cancer (BC) undergo RT during their cancer therapy (Baskar et al., 2012), and 95% of these patients experience radiation-induced dermatitis (RID), the most common complication of RT (Iacovelli et al., 2012). RID appears with a variety of symptoms in patients with BC, such as edema, pruritus, erythema, moist desquamation, ulceration, necrosis, and hemorrhage. RID can also induce significant skin pigmentation, pain, and reduced quality of life (QOL) and can even lead to delay in or early discontinuation of conventional cancer therapies such as RT and chemotherapy (Leventhal and Young, 2017).

Several studies have been conducted on possible strategies for reducing the occurrence of RID after RT in patients with BC; however, no standard preventive treatment for RID has been clearly demonstrated to be effective (Chan et al., 2014). Therefore, in clinical practice, skincare managements, such as gentle washing with mildly acidic or neutral soap and application of moisturizing creams, are routinely recommended for patients with BC during or after the course of RT, although there is insufficient evidence supporting their efficacy (Wong et al., 2013; Kong et al., 2016).

A water-in-oil type non-steroidal moisturizer cream containing hyaluronic acid as the main component showed significant efficacy in preventing and reducing RID compared to an emollient base cream in terms of ameliorating skin toxicity, burning sensations, and erythema in the irradiated area in patients with BC (Primavera et al., 2006). It was also reported to have wound regeneration and anti-inflammatory effects similar to natural steroids. Therefore, it is regarded as a topical-use barrier regimen for radiation lesions that is effective in preventing RID and relieving RT-related skin toxicities (Leonardi et al., 2008).

Jaungo (Shiunko in Chinese and Japanese), a traditional herbal ointment mainly composed of *Lithospermum erythrorhizon* Siebold & Zucc. [Borraginaceae; Lithospermi radix] and *Angelica sinensis* (Oliv.) Diels [Apiaceae/Umbelliferae; Angelica sinensis), is registered for use as a topical drug for dermatitis in Korea. The symptoms approved by the Korean Ministry of Food and Drug Safety for the use of Jaungo are rough skin, frostbite, heat rash, anus laceration, and skin inflammation. It is commonly used to treat skin injuries due to abrasions, frost, or burns. It is also widely used in clinical practice to prevent RID in cancer patients (Kong et al., 2016). Several major components of the two herbs, such as shikonin, ferulic acid, and decursin, have been shown to have therapeutic effects, including improved wound healing, granulation tissue formation, and re-epithelization through antibacterial and anti-inflammatory mechanism (Huang et al., 2004; Hsiao et al., 2012). A prior study showed that Jaungo reduced the incidence of RID and delayed the occurrence of grade 2 or higher RID compared to gentle washing with neutral soap without any safety issues in patients with BC (Kong et al., 2016). Case series have also reported that Jaungo had favorable effects on RID on the scalp of patients with malignant brain tumors (Hayashi and Sato, 2011). However, a comparative clinical trial of Jaungo and a water-in-oil-type non-steroidal moisturizer has not yet been conducted.

Considering the limited options for preventing RID and the results of previous studies, we designed this prospective, randomized, single-blind pilot study (CRIS registration number KCT0005971) to compare two topical agents commonly used for RID—Jaungo and the water-in-oil type non-steroidal moisturizer. This is the first randomized controlled trial (RCT) to evaluate the preventive efficacy of topically applied Jaungo and compare it with that of the water-in-oil type non-steroidal moisturizer in RID patients with BC.

## 2 Materials and methods

### 2.1 Patient eligibility

The patient eligibility criteria were as follows: pathologically confirmed invasive BC, having undergone breast-conserving surgery, and adjuvant RT planned after surgery. Patients with a history of RT for any reason, unhealed scars in the breasts, soft tissue disease, or uncontrolled diabetes mellitus with HbA1c ≥ 6.5% were excluded. Patients with a metastatic cancer stage were also excluded. This study was approved by the Institutional Review Board (IRB) of Kyung Hee University Hospital at Gangdong, Seoul, Republic of Korea (IRB number KHNMCOH-2020-12-001-002).

### 2.2 Study design

Between March 2021 and July 2022, 50 patients were prospectively enrolled and randomly allocated to the Jaungo group or the moisturizer group based on random numbers generated using a 2 × 2 randomized permuted block design by an independent statistician. The sample size was estimated based on the grade of Radiation Therapy Oncology Group (RTOG) (Cox, 1995) and National Cancer Institute (Trotti et al., 2000) for the RID reported in the relevant literature. The incidence rates that we estimated for the Jaungo and moisturizer groups were 0.467 and 0.09, respectively. The calculation formula of the sample size is described in the protocol paper of this study (Kim et al., 2021). The single-blind design was used for this study because the treatments could be distinguished by the participants. Therefore, the assessor who evaluates the progress of RID maintained a single-blind that did not know what treatment each participant received. All subjects visited nine times with a 3-day visit window—eight times during the course of RT after screening and once for a follow-up 2 weeks after the end of RT.

### 2.3 Study materials

Participants allocated to the intervention group, i.e., the Jaungo group, were instructed to apply Jaungo (Hanpoong Pharm & Foods t Corporation, Seoul, Republic of Korea; expiration date 02.14.2024). Jaungo is an external ointment consisting of *L. erythrorhizon*, Angelica sinensis, sesame oil, beeswax, and lard. The detailed compositions and the quality of control of Jaungo are presented in the Supplementary Material S1. The participants in the intervention group started applying Jaungo from the evening of the first day of RT, and applied it thinly on the lesion 1 cm wider than the entire irradiated skin area three times a day, at intervals of at least 4 h, during the entire RT period (5–7 weeks). The ointment should be applied thoroughly to surgical wounds, around the nipples, under the breast, and in the armpit incisions. If guide lines for RT were drawn on the chest, Jaungo was applied carefully so that the lines were not erased by the ointment. Participants allocated to the control group applied the moisturizer (X-derm^®^, Pharmbio Korea Corporation, Seoul, Republic of Korea; expiration date 04.23.2023) in the same manner. Owing to the possibility of bolus effect, neither agent was to be applied within 4 h after an RT session, and both were cleaned off from the irradiated region immediately before each session. Jaungo and the moisturizer were contained in individually packaged tubes, and each tube was stored at room temperature between 1 and 30° in an airtight container. If skin irritation, a local rash, or itching occurred, the application of the agents was stopped by the assessors. No other prophylactic topical agents for RID were allowed to be used in either group, but topical and intravenous antibiotics were allowed as rescue medicine.

### 2.4 Radiation therapy

All subjects underwent 3-dimensional conformal RT (3D-CRT) or intensity-modulated RT (IMRT) for 5–7 weeks with five fractions each week, and the tumor bed was treated with a biological equivalent dose (BED_10_) ≥ 60 Gy according to the National and International Guidelines. For participants who had risk factors for regional recurrence, such lymphovascular or axillary lymph node invasion, regional lymph node irradiation was also performed.

### 2.5 Outcome measurement

Information regarding demographic characteristics, including sex, age, height, weight, Eastern Cooperative Oncology Group (ECOG) performance status, final educational background, disease history, smoking and diabetes mellitus status, was collected. Information on clinical characteristics, such as breast volume, irradiation side (right or left), histology results and malignant tumor cell molecular subtype, cancer stage based on TNM staging, lymph node irradiation (Yes/No), and concurrent chemotherapy (Yes/No) was also recorded. For the assessment of outcomes, the severity and date of onset of RID, QOL, skin reaction symptoms, and pain intensity, including that of maximum pain related to RID, were evaluated based on the scale of Radiation Therapy Oncology Group (RTOG) criteria (Cox, 1995). Outcome measurements were conducted by a radiation oncologist maintaining blindness using the Catterall skin scoring profile (CSSP), (Catterall et al., 1971) the Korean version of Skindex-29 (Ahn et al., 2004), and the numeric rating scale (NRS).

The incidence rate of RID of RTOG grade ≥2 was evaluated as the primary outcome in this study. As secondary outcomes, the time to RID onset, incidence rate of maximum grade RID, CSSP, Skindex-29, and NRS scores, and adverse events (AEs) based on the Common Terminology Criteria for Adverse Events (CTCAE) version 5.0 were assessed at the start (visit 1) and end (visit 8) of RT and 2 weeks later (visit 9) to compare between the Jaungo and moisturizer groups (Freites-Martinez et al., 2021).

### 2.6 Statistical analysis

The characteristics of all participants are reported as mean and 95% confidence interval (95% CI) values for continuous variables or frequencies and percentages for categorical variables. The difference in incidence rate of RID of RTOG grade ≥2, the primary outcome, between the two groups was assessed using the Chi-square test. Among the secondary outcomes, the difference in incidence rate of maximum grade RID between groups was assessed using the Chi-square test, between time to RID onset using Kaplan-Meier analysis, and between CSSP, Skindex-29, and maximum NRS scores using the independent samples *t*-test or Mann–Whitney *U* test. The types, frequencies, and severity of AEs were also assessed; events that were determined to be caused by each intervention are separately analyzed and reported. All analyses were performed using SPSS version 25.0 (Chicago, IL, United States of America) based on the intention-to-treat (ITT) set. All tests were two-sided, and differences with a *p* value <0.05 were considered statistically significant.

## 3 Results

### 3.1 Study population

**FIGURE 1:**
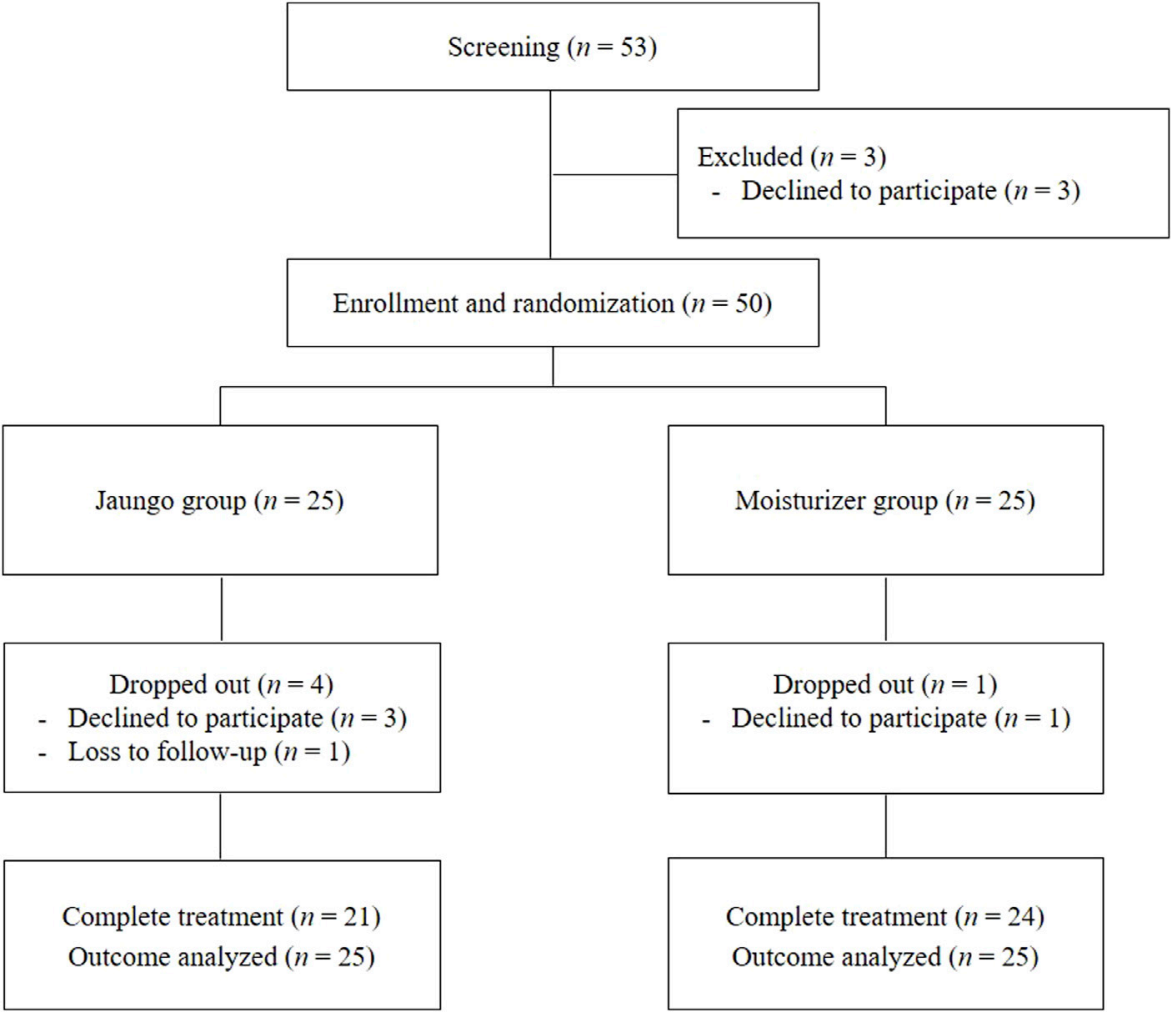
Flowchart of participant.

Forty-five of the 50 participants completed the study and applied Jaungo or the moisturizer. In both groups, the mean compliance rate was 98.8%, with no significant difference between groups (*p* = 0.672). Three participants in the Jaungo group and one in the moisturizer group refused to use topical agents during the study and declined to participate. One more participant in the Jaungo group was excluded from the analysis due to loss to follow-up. Therefore, 21 participants in the Jaungo group and 24 in the moisturizer group completed treatment, and all 50 participants were included in the efficacy and safety assessment based on ITT analysis (Figure 1).

### 3.2 The characteristics of participants

The characteristics of the participants are summarized in Table 1. All subjects were female, and mean age was 61.0 ± 10.3 years in the Jaungo group and 55.0 ± 9.2 years in the moisturizer group. Mean age in the Jaungo group was significantly higher than that in the moisturizer group (*p* = 0.034). The body mass index (BMI) was 25.0 ± 5.0 and 24.2 ± 3.3 kg/m^2^, respectively, with no significant differences in height, weight, or BMI. The mean breast volume calculated using the RT planning computer was 671.1 ± 316.2 versus 642.9 ± 321.8 cc, respectively. The number of participants who underwent concurrent chemotherapy during RT was five (20.0%) and three (12.0%) in each group, and all received taxol-based chemotherapy. There was no statistically significant difference (*p* = 0.702). The most common type of histology was invasive ductal carcinoma, followed by ductal carcinoma *in situ*. In both groups, there was one participant (4.0%) with confirmed triple-negative cancer cell molecular subtype, which is usually more aggressive than the estrogen receptor-/progesterone receptor-positive or human epidermal growth factor receptor-positive types (Pal et al., 2011). Regarding cancer stage based on TNM staging, most participants had stage 1 cancer (64.0% vs. 60.0%, respectively). There were no significant differences in demographic and clinical characteristics, except mean age, between the two groups.

**TABLE 1:** Demographic and clinical characteristics of study participants.

	Jaungo group	Moisturizer group	*p*-value
Age (years)	61.0 ± 10.3 (56.8, 65.3)	55.0 ± 9.2 (51.2, 58.8)	0.034*
Height (cm)	155.7 ± 6.9 (152.9, 158.5)	158.0 ± 5.6 (155.7, 160.3)	0.204
Weight (kg)	60.7 ± 12.0 (55.7, 65.6)	60.4 ± 8.5 (56.9, 63.9)	0.935
BMI (kg/m^2^)	25.0 ± 5.0 (23.0, 27.1)	24.2 ± 3.3 (22.9, 25.6)	0.504
ECOG performance status			
0/1	25/0	25/0	1.000
Smoking			
Yes/No	1/24	1/24	1.000
Diabetes mellitus			
Yes/No	1/24	3/22	0.609
Breast volume (cc)	671.1 ± 316.2 (552.6, 793.7)	642.9 ± 321.8 (528.3, 780.8)	0.678
Site			
Right/Left	18/7	17/8	0.758
Histology			
Invasive ductal carcinoma	14	15	0.774
Invasive lobular carcinoma	1	1	1.000
Encapsulated papillary carcinoma	3	2	0.500
Ductal carcinoma *in situ*	7	4	0.306
Myxoid liposarcoma	1	1	1.000
Stage			
0	7	4	0.306
1	16	15	0.771
2A	0	3	0.235
2B	1	2	1.000
3	1	1	1.000
Molecular subtype			
ER-positive	23	20	0.417
PR-positive	20	20	1.000
HER2-positive	5	5	1.000
Triple-negative	1	1	1.000
Lymph node irradiation			
Yes/No	5/20	4/21	0.713
Concurrent chemotherapy			
Yes/No	3/22	5/20	0.702

*p < 0.05.

SD, standard deviation; CI, confidence interval; BMI, body mass index; ECOG, eastern cooperative oncology group; HER2, human epidermal growth factor receptor 2; ER, estrogen receptor; PR, progesterone receptor.

### 3.3 Comparative efficacy of Jaungo and the moisturizer

**FIGURE 2:**
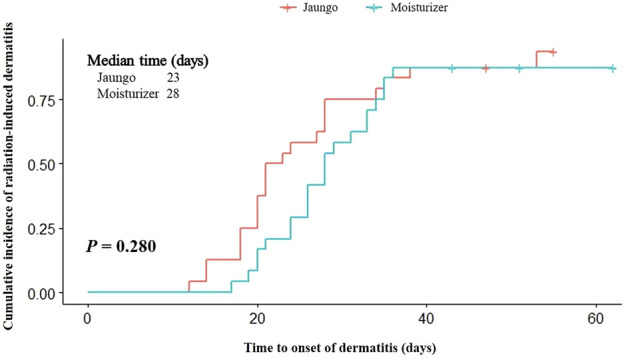
Kaplan-Meier analysis and log-rank test results for the time to radiation-induced dermatitis onset.

The outcome results are summarized in Table 2. The incidence rate of RID grade ≥2 was 24.0% (6 subjects) and 20.0% (5 subjects) in the Jaungo and moisturizer groups, respectively (*p* = 0.733). There were no subjects with RID over grade 2, and 12.0% (three subjects) and 16.0% (four subjects) did not experience RID during the entire trial period. There was no significant difference in the incidence of maximum grade RID between the two groups. The mean time to RID onset was 26.5 ± 11.8 (95% CI: 21.6, 31.5) versus 30.4 ± 10.3 (95% CI: 26.0, 34.7) days, respectively (*p* = 0.092). In addition, the cumulative incidence of RID, as assessed using the log-rank test, did not differ significantly between the groups (*p* = 0.280) (Figure 2). Among participants who received concurrent chemotherapy, the incidence rate was 20.0% (1 subject) and 33.3% (1 subject). Both were confirmed as grade 2 dermatitis, and there was no significant difference (*p* = 0.536).

**TABLE 2:** Analyses of primary and secondary outcomes.

	Jaungo group (n = 25)	Moisturizer group (n = 25)	*p*-value
Incidence rate of RID (%)			
RTOG grade ≥2	24.0	20.0	0.733
Maximum grade			0.890
2	24.0	20.0	
1	64.0	64.0	
0	12.0	16.0	
Time to RID onset (days)	26.5 ± 11.8 (21.6, 31.5)	30.4 ± 10.3 (26.0, 34.7)	0.092
CSSP score			
Visit 1	1.0 ± 0.0	1.0 ± 0.0	-
Visit 8	2.0 ± 0.7 (1.7, 2.3)	2.3 ± 0.7 (2.0, 2.6)	0.112
Visit 9	2.4 ± 1.4 (1.9, 3.0)	2.2 ± 0.7 (1.9, 2.5)	0.611
Skindex-29 scores			
Symptoms score			
Visit 1	31.5 ± 50.9 (10.6, 52.6)	43.0 ± 74.7 (12.1, 73.8)	0.496
Visit 8	126.5 ± 124.6 (75.1, 177.9)	201.4 ± 173.6 (129.7, 273.1)	0.087
Visit 9	213.8 ± 181.9 (138.7, 288.9)	131.6 ± 182.7 (56.2, 207.0)	0.088
Functioning score			
Visit 1	11.0 ± 44.5 (7.4, 29.7)	19.8 ± 46.5 (0.6, 39.0)	0.100
Visit 8	186.6 ± 271.5 (74.5, 298.7)	215.6 ± 243.2 (115.2, 316.0)	0.346
Visit 9	276.4 ± 354.1 (130.2, 422.6)	135.1 ± 284.1 (17.8, 252.4)	0.079
Emotion score			
Visit 1	34.4 ± 59.0 (10.1, 58.7)	44.2 ± 71.9 (14.5, 73.9)	0.967
Visit 8	159.0 ± 245.0 (57.9, 260.1)	150.0 ± 185.2 (73.5, 226.05)	0.526
Visit 9	255.2 ± 311.6 (126.6, 383.8)	123.6 ± 233.0 (31.6, 215.6)	0.074
NRS score			
Visit 1	0.48 ± 0.82 (0.14, 0.82)	0.28 ± 0.74 (−0.02, 0.58)	0.217
Visit 8	2.52 ± 2.43 (1.52, 3.52)	3.84 ± 3.24 (2.50, 5.18)	0.170
Visit 9	3.54 ± 3.19 (2.22, 4.86)	1.88 ± 2.88 (0.69, 3.07)	0.045*
Maximum NRS score	4.02 ± 3.02 (2.77, 5.27)	4.20 ± 3.40 (2.80, 5.60)	0.844
ECOG performance status			
Visit 1 (ECOG 0/1)	25/0	25/0	0.000
Visit 8 (ECOG 0/1)	25/0	23/2	0.245
Visit 9 (ECOG 0/1)	25/0	24/1	0.500

*p < 0.05.

RID, radiation-induced dermatitis; RTOG, radiation therapy oncology group; SD, standard deviation; CI, confidence interval; CSSP, catterall skin scoring profile; NRS, numeric rating scale; ECOG, eastern cooperative oncology group.

CSSP and Skindex-29 were used to assess the effects of RID on participant QOL. The CSSP scores at visit 1, 8, and 9 did not differ significantly between the two groups. The Skindex-29 total score (on three subscales—symptoms, functioning, and emotion) also showed no difference at any visit. However, when the Skindex-29 subscale scores were separately analyzed, there were significant differences between the groups with regard to the scores for the following items: ‘My skin condition burns or stings,’ and ‘My skin itches’ on the symptoms scale at visit 8, ‘My skin condition affects how well I sleep’ at visit 8 and ‘I tend to stay at home because of my skin condition’ at visit 9 on the functioning scale, and ‘My skin condition makes me feel depressed’ at visit 9 on the emotion scale. The scores for 29 Skindex-29 items are presented in the Supplementary Material S2. The RID-related pain score assessed using the NRS was higher in the Jaungo group at visit 9, 2 weeks after the end of RT (3.54 ± 3.19 [2.22, 4.86] versus 1.88 ± 2.88 [0.69, 3.07], *p* = 0.045), but pain scores at other visits and maximum pain score did not differ significantly between the groups.

### 3.4 Adverse events

In the safety analysis, only one participant in the moisturizer group had mild fever and grade 1 insomnia based on CTCAE version 5.0. Both symptoms were evaluated as likely unrelated to the topical agent and resolved without discontinuing treatment or RT. Additionally, there were no severe AEs during the entire trial period.

## 4 Discussion

RID is one of the most common side-effects of RT. Patients with RID can experience various symptoms, such as edema, pruritis, erythema, moist desquamation, ulceration, necrosis, and hemorrhage in the irradiated regions, and have delayed or discontinued conventional cancer therapies because of the events (Leventhal and Young, 2017; Iacovelli et al., 2020). According to previous reports, up to 90% of patients with BC suffer from RT-induced skin reactions (Ding et al., 2018), and one out of three patients develops chronic RID, which can appear up to 10 years after the end of RT (Leventhal and Young, 2017). The incidence and severity of RID are affected by various risk factors such as age, BMI, smoking, breast volume, RT dose, and concurrent chemotherapy (Ramseier et al., 2020; Xie et al., 2021). As such, RID accounts for a high proportion of AEs in patients with BC who have several risk factors, but there is an absence of sufficient evidence to support the use of clinically applied agents in preventing RID in patients with BC. Therefore, there are currently no standard treatments or guidelines for RID prevention. In clinical practice, topical management, including the use of moisturizers, topical steroidal therapies, gentle washing with mildly acidic or neutral soaps, and dressing, is generally used for patients with BC and RID, despite the evidence of their efficacy being debatable (Wong et al., 2013; Kong et al., 2016).

There are various risk factors for RID in patients with BC. Regarding therapy-related factors, the dose and duration of RT and concurrence with other conventional cancer therapies have been reported to affect the incidence of RID on irradiated skin. In addition, high breast volume, older age, high BMI, poor ECOG performance status, smoking, and alcohol consumption are patient-related factors associated with the early development of RID (Ramseier et al., 2020; Xie et al., 2021). Considering this background, no demographic and clinical characteristics of participants, including BMI, ECOG performance status, smoking, breast volume, and concurrent chemotherapy, except mean age, showed significant differences between the Jaungo and moisturizer groups in this study. The dose and duration of RT were determined according to the radiation oncologist’s prescription based on the National and International Guidelines, and the minimum dose in this study was BED_10_ ≥ 60 Gy. The mean age in the Jaungo group was significantly higher than that in the moisturizer group. Based on the prior background, since it has been reported that the incidence of RID increases with age, participants who used Jaungo could be expected to be at a higher risk of RID incidence than those who used the moisturizer. However, our results showed no significant differences in the incidence rate of RID grade ≥2, maximum grade, mean time to RID onset, or cumulative incidence of RID between the two groups. These outcomes support the potential benefit of Jaungo in preventing RID in patients with BC independently of risk factors, and also indicate that it is comparable to the water-in-oil type non-steroidal moisturizer.

The water-in-oil type non-steroidal moisturizer is one of the topical agents clinically applied for the prevention of RID and contains hyaluronic acid, shea butter, glycyrrhetinic acid, Vitis vinifera extract, and telmesteine (Primavera et al., 2006). All of these components are widely used as moisturizing agents that can retain water under the skin barrier. In particular, the main component, hyaluronic acid, has hygroscopic properties and has been used to treat wound regeneration (Abramovits et al., 2006). Other components have also been reported to have anti-inflammatory properties, and glycyrrhetinic acid can even act as a natural steroid (Kroes et al., 1997). In a prior study, the moisturizer was reported to be safe and effective in preventing and minimizing RT-related skin symptoms in patients with BC. It showed statistically significant efficacy against the maximum severity of skin toxicity (*p* < 0.0001), burning sensations (*p* = 0.039), and moist desquamation within radiation lesions (*p* = 0.02) compared to the other topical agent without the key components (Abramovits et al., 2006). Therefore, the moisturizer is regarded as a clinical option for preventing RID in cancer patients. However, because there is still insufficient evidence supporting the preventive effect of the moisturizer on RID, it is not a standard recommended treatment for RID prevention, and till date there are no standard guidelines for preventing RID in cancer patients so far.

Jaungo (Shiunko in Chinese and Japanese) is a traditional herbal ointment standardized according to the Korean Pharmacopeia. Its main components are Lithspermi radix and Angelica sinensis. The Korea Food and Drug Safety Administration (KFDA) has approved its use as a topical drug for dermatitis. The active ingredients have been identified as shikonin, acetylshikonin, ferulic acid, and decursin, which have been reported to promote soft tissue formation, wound regeneration, and skin healing due to their antibacterial and anti-inflammatory properties (Hsiao et al., 2012; Kong et al., 2016; Huang et al., 2006). Based on the effects of these components, *L. erythrorhizon* and Angelica sinensis improve pruritus and skin inflammation as symptoms of dermatitis by blocking the release of histamine and the activation of pro-inflammatory cytokines (Kim et al., 2007; Kong et al., 2016; Lee et al., 2016). According to previous studies, Jaungo has been evaluated for potential efficacy through proteomics and bioinformatics approach using a cell model system for the wound healing process (Chak et al., 2013). Jaungo showed anti-inflammatory and skin regeneration effects by reducing Immunoglobulin E (IgE), thymus and activation-regulated chemokine (TARC), and interferon-gamma (IFNγ) and inhibiting production of eotaxin for dermatitis. In addition, it has been reported to alleviate skin inflammation by inducing proteins of fibroblast such as peroxiredoxin (PRDX)2, PRDX4, superoxide dismutase C (SODC), and glutathione S-transferase P (GSTP)1 to show antioxidant effects (Chak et al., 2013; Choi et al., 2017). Therefore, Jaungo has been clinically applied to treat a variety of skin symptoms, including not only general dermatitis but also RID in cancer patients. Indeed, according to a recent case series study, Jaungo showed preventive efficacy against scalp RID in patients with malignant brain tumors who underwent RT and was regarded to improve RID-related symptoms including redness, burning pain, itching, and erosion on the irradiated skin (Hayashi and Sato, 2011). In addition, Jaungo also reduced the incidence rate of RID grade ≥2 (46.7% versus 78.6%) and grade 3 (20.0% versus 50.0%) without any severe safety issue in BC patients compared to general skin management including gentle washing with neutral soap, although the differences were not statistically significant (Kong et al., 2016). Therefore, in this study, which was conducted using the efficacy comparison design, the finding that Jaungo showed no statistically significant difference in RID-related incidence, QOL, and pain compared to the moisturizer can be used as more substantial evidence for the preventive effects of Jaungo. In addition, based on previous studies, Jaungo can be applied not only to patients with BC but also to various cancer patient groups receiving RT, including patients with brain tumor. Given the high prevalence of RID and the limited options for preventing it in patients with BC, we conducted this prospective, randomized, controlled, single-blind pilot study that compared the preventive efficacy of Jaungo and a water-in-oil type non-steroidal moisturizer. The results showed no significant differences between the two topical agents in terms of the incidence rates of RID and RID-related symptoms. In addition, there were no AEs in subjects who applied Jaungo during the entire period of RT. However, two patients who declined to participate in the Jaungo group were repulsed by the smell of the ointment. As this is caused by the main components of Jaungo, *L. erythrorhizon* and Angelica sinensis, it is considered that a detailed explanation for patients in this regard will be needed in future studies.

This study provides evidence that Jaungo has comparable efficacy to the moisturizer for RID in patients with BC. However, it has some limitations. First, this trial was a pilot study with a small sample size. Although the number of patients in each group was estimated statistically based on previous research, further larger sample size RCTs are needed. Second, this study was conducted in a single-blind manner, which means that the subjects knew whether they applied Jaungo or the moisturizer. This may have been a confounding factor; therefore, placebo-controlled trials are required to ensure double-blinding. Despite these limitations, this is the first pilot RCT to evaluate the comparative efficacy and safety of Jaungo and the moisturizer for RID in patients with BC. We believe that the results of this study will encourage further well-designed, double-blind, large-scale RCTs on preventing RID related to conventional cancer therapies.

## 5 Conclusion

Jaungo, a traditional herbal ointment which mainly consists of *L. erythrorhizon* and Angelica sinensis extracts, has comparable efficacy in preventing RID in patients with BC to a water-in-oil-type non-steroidal moisturizer cream currently used in clinical practice. However, large-sample, double-blind RCTs with placebo-controlled are required to fully define the efficacy and safety of Jaungo in preventing RID in patients with BC. (Catterall et al., 1971).

## Data Availability

The original contributions presented in the study are included in the article/Supplementary Material, further inquiries can be directed to the corresponding authors.
