# Corrigendum: Comparative efficacy of Jaungo, a traditional herbal ointment, and a water-in-oil type non-steroidal moisturizer for radiation-induced dermatitis in patients with breast cancer: a prospective, randomized, single-blind, pilot study

**DOI:** 10.3389/fphar.2023.1326887

**Published:** 2023-11-28

**Authors:** Eun Hye Kim, Su Bin Park, Hayun Jin, Weon Kuu Chung, Seong Woo Yoon

**Affiliations:** ^1^ Department of Clinical Korean Medicine, College of Korean Medicine, Graduate School, Kyung Hee University, Seoul, Republic of Korea; ^2^ Department of Korean Internal Medicine, Kyung Hee University Hospital at Gangdong, Seoul, Republic of Korea; ^3^ Department of Radiation Oncology, Kyung Hee University at Gangdong, Seoul, Republic of Korea

**Keywords:** Jaungo, Shiunko, radiation-induced dermatitis, traditional medicine, breast cancer

In the published article, there was an error in **Affiliation 1**. Instead of “Department of Clinical Korean Medicine, Graduate School, Kyung Hee University, Seoul, Republic of Korea,” it should be “Department of Clinical Korean Medicine, College of Korean Medicine, Graduate School, Kyung Hee University, Seoul, Republic of Korea.”

The authors apologize for this error and state that this does not change the scientific conclusions of the article in any way. The original article has been updated.

