# Supplementary material for: Comparative efficacy of Jaungo, a traditional herbal ointment, and a water-in-oil type non-steroidal moisturizer for radiation-induced dermatitis in patients with breast cancer: a prospective, randomized, single-blind, pilot study
Source: Front Pharmacol. 2023 Jul 3;14:1216668. doi: 10.3389/fphar.2023.1216668 (PMC10353018; doi:10.3389/fphar.2023.1216668)
Supplement: Supplementary file 1 [file DataSheet1.docx]

Supplementary Material

Comparative Efficacy of Jaungo, a Traditional Herbal Ointment, and a Water-in-Oil Type Non-Steroidal Moisturizer for Radiation-induced Dermatitis in Patients with Breast Cancer: A Prospective, Randomized, Single-Blind, Pilot Study

Eun Hye Kim^1^, KMD, MS, Su Bin Park^2^, KMD, MS, Hayun Jin^2^, KMD, BS, Weon Kuu Chung^3*^, MD, PhD, Seong Woo Yoon^2*^, KMD, PhD

^1^Department of Clinical Korean Medicine, Graduate School, Kyung Hee University, Seoul, Republic of Korea

^2^ Department of Korean Internal Medicine, Kyung Hee University Hospital at Gangdong, Seoul, Republic of Korea

^3^Department of Radiation Oncology, Kyung Hee University of Gangdong, Seoul, Republic of Korea

*** Correspondence:**Seong Woo Yoon, stepano212@hanmail.net; Weon Kuu Chung, wkchung16@gmail.com

# Supplementary Material S1. The detailed compositions of Jaungo

**1 Material composition of Jaungo**

1g of Jaungo (Shiunko in Chinese and Japanese) consists of *Lithospermum erythrorhizon* Siebold & Zucc. [Borraginaceae; Lithospermi radix] (72.7mg), *Angelica sinensis* (Oliv.) Diels [Apiaceae/Umbelliferae; Angelica sinensis) (60.6mg), sesame oil (606.1mg), beeswax (202.7mg or 242.4mg), and lard (18.2mg). The main compositions of Jaungo are *Lithospermi Radix* and *Angelica sinensis*. Among the others, lard is used as a base layer, and sesame oil and beeswax are added as additives.

**2 Dosage of active ingredients**

Quality control is performed by dosage of active ingredients extracted from the two herbs. 0.07mg of shikonin extracted from *Lithospermi Radix* and 3.6mg of decursin extracted from *Angelica sinensis* have to be confirmed per 1g of Jaungo.

**3 Preparation method**

Heat sesame oil for 1-2 hours to remove moisture and drop it into water to make it a condensed spherical base layer. Add beeswax (242.4mg in summer, 202.7mg in winter) and lard to melt, and then, while heating at 140 degrees, add thinly sliced *Angelica sinensis* and mix all. When it turns dark brown, add thinly sliced *Lithospermi Radix*, check boiling, and stir 2-3 times. When it turns bright purple-red (clear magenta color), turn off the heat, filter it with cotton patch, and store it in the refrigerator. After the process, cut it into even shapes and use it. When manufacturing Jaungo, it is prepared in units of 1kg and then subdivided for use.

# Supplementary Material S2. Skindex-29 scores in the Jaungo and moisturizer groups

|  | **Jaungo group (n = 25)** | **Moisturizer group (n = 25)** | **P-value** |
| --- | --- | --- | --- |
| *Symptoms scale* |  |  |  |
| My skin hurts |  |  |  |
| Visit 1 | 8.6 ± 16.4 (1.8, 15.4) | 4.8 ± 11.6 (0.0, 9.6) | 0.349 |
| Visit 8 | 27.2 ± 31.3 (14.3, 40.1) | 40.0 ± 32.8 (26.5, 53.5) | 0.121 |
| Visit 9 | 37.2 ± 33.2 (23.5, 50.9) | 20.4 ± 33.0 (6.8, 34.0) | 0.051 |
| My skin burns or stings |  |  |  |
| Visit 1 | 0.8 ± 2.8 (-0.3, 1.9) | 2.0 ± 4.1 (0.3, 3.7) | 0.226 |
| Visit 8 | 21.5 ± 27.3 (10.2, 32.8) | 41.0 ± 35.8 (26.2, 55.8) | 0.035* |
| Visit 9 | 39.8 ± 35.4 (25.2, 54.4) | 21.6 ± 33.6 (7.7, 35.5) | 0.064 |
| My skin itches |  |  |  |
| Visit 1 | 1.4 ± 3.4 (0.0, 2.8) | 7.7 ± 17.4 (0.5, 14.8) | 0.381 |
| Visit 8 | 14.2 ± 22.4 (4.9, 23.5) | 36.8 ± 32.6 (23.3, 50.3) | 0.011* |
| Visit 9 | 36.4 ± 30.1 (24.0, 48.8) | 22.8 ± 29.2 (10.7, 34.9) | 0.097 |
| Water bothers my skin (bathing, washing hands) |  |  |  |
| Visit 1 | 6.4 ± 15.0 (0.2, 12.6) | 3.2 ± 10.3 (-1.0, 7.4) | 0.622 |
| Visit 8 | 21. 2 ± 33.6 (7.3, 35.1) | 25.2 ± 31.2 (12.3, 38.1) | 0.352 |
| Visit 9 | 31.6 ± 37.3 (16.2, 47.0) | 20.8 ± 29.6 (8.6, 33.0) | 0.354 |
| My skin is irritated |  |  |  |
| Visit 1 | 5.2 ± 15.3 (-1.1, 11.5) | 11.7 ± 27.7 (0.3, 23.2) | 0.315 |
| Visit 8 | 24.0 ± 34.8 (9.7, 38.3) | 29.6 ± 33.6 (15.7, 43.5) | 0.458 |
| Visit 9 | 33.2 ± 36.5 (17.2, 47.3) | 18.8 ± 28.5 (7.0, 30.6) | 0.284 |
| My skin is sensitive |  |  |  |
| Visit 1 | 9.2 ± 20.0 (1.0, 17.4) | 10.8 ± 25.9 (0.1, 21.5) | 0.586 |
| Visit 8 | 17.6 ± 25.2 (7.2, 28.0) | 25.6 ± 33.7 (11.7, 39.5) | 0.344 |
| Visit 9 | 28.2 ± 33.5 (14.4, 42.0) | 14.8 ± 29.3 (2.7, 26.9) | 0.095 |
| My skin bleeds |  |  |  |
| Visit 1 | 0.0 ± 0.0 (0.0, 0.0) | 2.8 ± 14.0 (-3.0, 8.6) | 0.317 |
| Visit 8 | 0.8 ± 2.8 (-0.3, 1.9) | 3.2 ± 14.1 (-2.6, 9.0) | 0.967 |
| Visit 9 | 8.4 ± 22.5 (-0.9, 17.7) | 12.4 ± 27.1 (1.2, 23.6) | 0.946 |
| Total symptoms scale score |  |  |  |
| Visit 1 | 31.6 ± 50.9 (10.6, 52.6) | 43.0 ± 74.7 (12.1, 73.8) | 0.709 |
| Visit 8 | 126.5 ± 124.6 (75.1, 177.9) | 201.4 ± 173.6 (129.7, 273.1) | 0.087 |
| Visit 9 | 213.8 ± 181.9 (138.7, 288.9) | 131.6 ± 182.7 (56.2, 207.0) | 0.088 |
|  |  |  |  |
| *Functioning scale* |  |  |  |
| My skin condition affects how well I sleep |  |  |  |
| Visit 1 | 0.4 ± 2.0 (-0.4, 1.2) | 3.2 ± 10.7 (-1.2, 7.6) | 0.284 |
| Visit 8 | 12.0 ± 24.1 (2.0, 22.0) | 28.8 ± 34.6 (14.5, 43.1) | 0.034* |
| Visit 9 | 29.0 ± 34.4 (14.8, 43.2) | 17.2 ± 30.6 (4.6, 30.6) | 0.093 |
| My skin condition makes it hard to work or practice hobbies |  |  |  |
| Visit 1 | 0.4 ± 2.0 (-0.4, 1.2) | 2.4 ± 7.2 (-0.6, 5.4) | 0.284 |
| Visit 8 | 16.8 ± 24.8 (6.6, 27.0) | 30.4 ± 33.1 (16.7, 44.1) | 0.091 |
| Visit 9 | 26.4 ± 33.5 (12.6, 40.2) | 15.5 ± 27.4 (4.2, 26.8) | 0.270 |
| My skin condition affects my social life |  |  |  |
| Visit 1 | 0.6 ± 2.2 (-0.3, 1.5) | 0.4 ± 2.0 (-0.4, 1.2) | 0.572 |
| Visit 8 | 10.0 ± 18.9 (2.2, 17.8) | 20.4 ± 26.5 (9.4, 31.4) | 0.083 |
| Visit 9 | 22.8 ± 33.8 (8.8, 36.8) | 10.0 ± 23.3 (0.4, 19.6) | 0.163 |
| I tend to stay at home because of my skin condition |  |  |  |
| Visit 1 | 2.0 ± 10.0 (-2.1, 6.1) | 2.4 ± 10.1 (-1.8, 6.6) | 0.572 |
| Visit 8 | 21.0 ± 32.9 (7.4, 34.6) | 31.6 ± 38.7 (15.6, 47.6) | 0.295 |
| Visit 9 | 33.2 ± 38.9 (17.1, 49.3) | 14.0 ± 31.2 (1.1, 26.9) | 0.045* |
| My skin condition affects how close I can be with those I love |  |  |  |
| Visit 1 | 0.4 ± 2.0 (-0.4, 1.2) | 0.4 ± 2.0 (-0.4, 1.2) | 1.000 |
| Visit 8 | 16.8 ± 31.5 (3.8, 29.8) | 13.6 ± 23.1 (4.1, 23.1) | 0.954 |
| Visit 9 | 17.8 ± 32.0 (4.6, 31.0) | 10.8 ± 25.2 (0.4, 21.2) | 0.244 |
| I tend to do things by myself because of my skin condition |  |  |  |
| Visit 1 | 2.0 ± 10.0 (-2.1 ,6.1) | 2.4 ± 10.1 (-1.8, 6.6) | 0.572 |
| Visit 8 | 16.0 ± 26.6 (5.0, 27.0) | 10.8 ± 17.1 (3.8, 17.8) | 0.854 |
| Visit 9 | 20.0 ± 33.4 (6.2, 33.8) | 8.4 ± 21.3 (-0.4, 17.2) | 0.166 |
| My skin condition makes showing affection difficult |  |  |  |
| Visit 1 | 0.4 ± 2.0 (-0.4, 1.2) | 0.4 ± 2.0 (-0.4, 1.2) | 1.000 |
| Visit 8 | 17.2 ± 27.8 (5.7, 28.7) | 8.8 ± 21.7 (-0.1, 17.7) | 0.326 |
| Visit 9 | 23.2 ± 39.1 (7.0, 39.4) | 8.4 ± 22.5 (-0.9, 17.7) | 0.140 |
| My skin condition affects my interactions with others |  |  |  |
| Visit 1 | 0.4 ± 2.0 (-0.4, 1.2) | 18.8 ± 33.3 (5.0, 32.6) | 0.317 |
| Visit 8 | 18.8 ± 33.3 (5.0, 32.6) | 10.8 ± 21.0 (2.1, 19.5) | 0.590 |
| Visit 9 | 15.0 ± 29.7 (2.8, 27.2) | 7.6 ± 20.1 (-0.7, 15.9) | 0.195 |
| My skin condition is a problem for the people I love |  |  |  |
| Visit 1 | 1.2 ± 6.0 (-1.3, 3.7) | 2.4 ± 10.1 (-1.8 ,6.6) | 0.556 |
| Visit 8 | 12.8 ± 23.0 (3.3, 22.3) | 19.2 ± 24.3 (9.2, 29.2) | 0.200 |
| Visit 9 | 25.0 ± 34.4 (10.8, 39.2) | 13.2 ± 30.8 (0.5, 25.9) | 0.064 |
| My skin condition affects my desire to be with people |  |  |  |
| Visit 1 | 0.0 ± 0.0 (0.0, 0.0) | 0.0 ± 0.0 (0.0, 0.0) | 1.000 |
| Visit 8 | 12.0 ± 22.7 (2.6 ,21.4) | 8.8 ± 17.9 (1.4, 16.2) | 0.696 |
| Visit 9 | 18.0 ± 35.0 (3.6, 32.4) | 8.0 ± 21.4 (-0.8, 16.8) | 0.272 |
| My skin condition interferes with my sex life |  |  |  |
| Visit 1 | 0.8 ± 4.0 (-0.9, 2.5) | 2.0 ± 10.0 (-2.1, 6.1) | 0.977 |
| Visit 8 | 12.4 ± 26.7 (1.4, 23.4) | 9.2 ± 22.2 (0.1, 18.3) | 0.523 |
| Visit 9 | 20.0 ± 33.4 (6.2, 33.8) | 7.2 ± 19.0 (-0.7, 15.1) | 0.154 |
| My skin condition makes me tired |  |  |  |
| Visit 1 | 2.8 ± 14.0 (-3.0 ,8.6) | 3.4 ± 11.4 (-1.4, 8.1) | 0.332 |
| Visit 8 | 20.8 ± 32.3 (7.5, 34.1) | 23.2 ± 30.2 (10.7, 35.7) | 0.600 |
| Visit 9 | 26.0 ± 35.1 (11.5, 40.5) | 14.8 ± 29.0 (2.8, 26.7) | 0.215 |
| Total functioning scale score |  |  |  |
| Visit 1 | 11.0 ± 44.5 (-7.4, 29.7) | 19.8 ± 46.5 (0.6, 39.0) | 0.100 |
| Visit 8 | 186.6 ± 271.5 (74.5, 298.7) | 215.6 ± 243.2 (115.2, 316.0) | 0.346 |
| Visit 9 | 276.4 ± 354.1 (130.2, 422.6) | 135.1 ± 284.1 (17.8, 252.4) | 0.079 |
|  |  |  |  |
| *Emotion scale* |  |  |  |
| I worry that my skin condition may be serious |  |  |  |
| Visit 1 | 4.0 ± 14.1 (-1.8, 9.8) | 5.2 ± 18.5 (-2.4, 12.8) | 0.449 |
| Visit 8 | 16.0 ± 23.8 (6.2, 25.8) | 29.6 ± 35.4 (15.0, 44.2) | 0.249 |
| Visit 9 | 37.6 ± 38.5 (21.7, 53.5) | 18.0 ± 30.1 (5.6, 30.4) | 0.058 |
| My skin condition makes me feel depressed |  |  |  |
| Visit 1 | 3.2 ± 14.1 (-2.6, 9.0) | 2.8 ± 10.6 (-1.6, 7.2) | 1.000 |
| Visit 8 | 19.6 ± 29.4 (7.5, 31.7) | 22.4 ± 29.1 (10.4, 34.4) | 0.480 |
| Visit 9 | 36.4 ± 39.3 (20.2, 52.6) | 12.8 ± 25.7 (2.2, 23.4) | 0.014* |
| I worry about getting scars from my skin condition |  |  |  |
| Visit 1 | 10.4 ± 25.2 (0.0, 20.8) | 14.8 ± 29.9 (2.5, 27.1) | 0.540 |
| Visit 8 | 25.0 ± 31.9 (11.8, 38.2) | 23.2 ± 31.3 (10.3, 36.1) | 0.822 |
| Visit 9 | 36.4 ± 38.0 (20.7, 52.1) | 18.4 ± 29.0 (6.4, 30.4) | 0.087 |
| I am ashamed of my skin condition |  |  |  |
| Visit 1 | 1.2 ± 6.0 (-1.3, 3.7) | 0.4 ± 2.0 (-0.4, 1.2) | 0.977 |
| Visit 8 | 14.4 ± 27.2 (3.2, 25.6) | 12.0 ± 25.7 (1.4, 22.6) | 0.451 |
| Visit 9 | 19.4 ± 35.8 (4.6, 34.2) | 9.6 ± 21.9 (0.6, 18.6) | 0.296 |
| I worry that my skin condition may get worse |  |  |  |
| Visit 1 | 14.0 ± 21.6 (5.1, 22.9) | 13.8 ± 25.9 (3.1, 24.5) | 0.937 |
| Visit 8 | 24.4 ± 34.7 (10.1, 38.7) | 12.4 ± 18.8 (4.7, 20.1) | 0.641 |
| Visit 9 | 28.8 ± 38.4 (12.9, 44.7) | 16.0 ± 27.2 (4.8, 27.2) | 0.173 |
| I am angry about my skin condition |  |  |  |
| Visit 1 | 1.2 ± 6.0 (-1.3, 3.7) | 1.2 ± 4.4 (-0.6, 3.0) | 0.588 |
| Visit 8 | 13.6 ± 25.8 (3.0, 24.2) | 8.8 ± 18.1 (1.3, 16.3) | 0.548 |
| Visit 9 | 20.0 ± 28.0 (8.4, 31.6) | 12.0 ± 26.3 (1.1, 22.9) | 0.158 |
| I am embarrassed by my skin condition |  |  |  |
| Visit 1 | 0.0 ± 0.0 (0.0, 0.0) | 0.0 ± 0.0 (0.0, 0.0) | 1.000 |
| Visit 8 | 15.2 ± 30.0 (2.8, 27.6) | 10.0 ± 20.8 (1.4, 18.6) | 0.835 |
| Visit 9 | 17.0 ± 30.7 (4.3, 29.7) | 10.4 ± 27.0 (-0.7, 21.5) | 0.234 |
| I am frustrated by my skin condition |  |  |  |
| Visit 1 | 0.0 ± 0.0 (0.0, 0.0) | 0.4 ± 2.0 (-0.4, 1.2) | 0.317 |
| Visit 8 | 10.8 ± 23.1 (1.3, 20.3) | 8.0 ± 16.1 (1.4, 14.6) | 0.943 |
| Visit 9 | 18.4 ± 29.3 (6.3, 30.5) | 6.4 ± 19.6 (-1.7, 14.5) | 0.059 |
| I am humiliated by my skin condition |  |  |  |
| Visit 1 | 0.2 ± 1.0 (-0.2, 0.6) | 0.4 ± 2.0 (-0.4, 1.2) | 0.977 |
| Visit 8 | 10.0 ± 18.7 (2.3, 17.7) | 6.4 ± 15.5 (0.0, 12.8) | 0.476 |
| Visit 9 | 18.4 ± 35.0 (4.0, 32.8) | 4.8 ± 15.3 (-1.5, 11.1) | 0.134 |
| I am annoyed by my skin condition |  |  |  |
| Visit 1 | 0.2 ± 1.0 (-0.2, 0.6) | 5.2 ± 13.9 (-0.5, 10.9) | 0.071 |
| Visit 8 | 10.0 ± 23.5 (0.3, 19.7) | 17.2 ± 27.9 (5.7, 28.7) | 0.412 |
| Visit 9 | 22.8 ± 36.1 (7.9, 37.7) | 15.2 ± 30.6 (2.6, 27.8) | 0.369 |
| Total emotion scale score |  |  |  |
| Visit 1 | 34.4 ± 59.0 (10.1, 58.7) | 44.2 ± 71.9 (14.5, 73.9) | 0.967 |
| Visit 8 | 159.0 ± 245.0 (57.9, 260.1) | 150.0 ± 185.2 (73.5, 226.5) | 0.526 |
| Visit 9 | 255.2 ± 311.6 (126.6, 383.8) | 123.6 ± 223.0 (31.6, 215.6) | 0.074 |

^*^*P* < 0.05

Data are presented as mean ± SD (95% CI) values.
